# Editorial: Venom Peptides: A Rich Combinatorial Library for Drug Development

**DOI:** 10.3389/fmolb.2022.924023

**Published:** 2022-05-13

**Authors:** Fernanda C. Cardoso, Denis Servent, Maria Elena de Lima

**Affiliations:** ^1^ Institute for Molecular Bioscience, The University of Queensland, Brisbane, QLD, Australia; ^2^ CEA, Département Médicaments et Technologies pour La Santé (DMTS), SIMoS, Université Paris-Saclay, Gif-sur-Yvette, France; ^3^ Programa de Pós Graduação em Medicina e Biomedicina, Santa Casa de Belo Horizonte, Belo Horizonte, Brazil; ^4^ Departamento de Bioquímica e Imunologia, Universidade Federal de Minas Gerais, Belo Horizonte, Brazil

**Keywords:** venom peptide, structure-function activity, drug developement, pharmacological tools, novel therapeutic agents

Venom peptides are exceptional natural molecules in which bioactivities led to venom-derived drugs such as Captopril to treat hypertension (Ferreira et al., 1970; Cushman and Ondetti, 1999), Ziconotide to treat chronic pain (Miljanich, 2004) and Exenatide to treat type-2 diabetes (Furman, 2012). Their proved translation into new therapeutics have attracted researchers seeking molecular tools and drugs for a variety of diseases from neurological disorders to cancer, autoimmunity and infection (Cardoso, 2020). In addition, venom peptides have been studied as pesticides which led to the development of the bioinsecticide Spear comprising a spider peptide (Chong et al., 2007). In this Research Topic in Frontiers of Molecular Biosciences, we captured state-of-the-art research in venom peptides through eight original research papers and five reviews. From spider, snake, scorpion and cone snail venoms applied in venomics and structure-function studies to the pre-clinical efficacy of venom-derived peptides, we navigate through exceptional research findings in the field of venom peptides and their applications into novel pharmacological and biotechnological tools.

Modern venom peptide discovery utilizes venom gland transcriptomics to unravel venom components and their molecular diversity (Ducancel et al., 2014). In Chase et al., this approach was applied to integrate venom peptides libraries from Turridae marine snails to better understand phylogenetic relationships and to identify novel bio-actives. Their findings support the conventional polyphyletic origins of this marine snail family and describes the new genus *Purpuraturris*. Novel venom peptides were identified and are under consideration for studies of characterization and therapeutic potentials. In another study, Kuhn-Nentwig et al. applied transcriptomics to explore linear peptides in modern spider venoms, a neglected group of membrane active peptides with cytolytic properties contributing to venom activity. These linear peptides were identified only in Ctenidae, Lycosidae, Oxyopidae, Pisauridae, and Zodariidae out of 23 families of spiders studied. Interestingly, in Lycosidae, Oxyopidae, and in the genus *Cupiennius*, these peptides are highly expressed, indicating functional importance in venom activity.

Linear venom peptides from ant (Touchard et al., 2016; Schifano and Caputo, 2021), spider (Melo-Braga et al., 2020), bee (Memariani and Memariani, 2021) and wasp (Dos Santos Cabrera et al., 2019) venoms have been investigated for antimicrobial properties against superbugs. These often comprise cationic, anionic or amphipathic peptides and display strong activity to penetrate cell membranes (Yacoub et al., 2020). Reis et al. investigated an optimized antimicrobial peptide named LyeTxI-b which is a derivative of LyeTxI isolated from the spider *Lycosa erythrognatha*. LyeTxI-*b* had higher affinity for anionic lipids and superior potency against *Staphylococcus aureus*. From the venom of the scorpion Tityus stigmurus, Melo et al. isolated a multifunctional anionic linear peptide displaying *Fe* ^*2+*^ *and Cu* ^*2+*^ chelating properties with potential as novel drugs for acute and chronic intoxication, neurodegeneration, haematological and cardiovascular diseases and cancer. Wasps are rich in mastoparans, another group multifunctional linear cationic peptides with therapeutic properties, as revised in Santana et al. These unique peptides have a wide variety of biological effects, including mast cell degranulation, activation of protein G, phospholipase A2, C, and D activation, serotonin and insulin release, antimicrobial, hemolytic, and anticancer activities.

Inhibitory cystine knot (ICK) peptides are predominant in spider and cone snail venoms and display unique therapeutic potential by modulating ion channels and receptors (Cardoso and Lewi, 2018; Cardoso and Lewi, 2019; Cardoso, 2020; Cardoso et al., 2021). The spider ICK peptide PnTx-2-6 was isolated from the Brazilian wandering spider *Phoneutria nigriventer* as reviewed by Silva et al. In clinical cases and pre-clinical studies, PnTx-2-6 induced priapism occurring via NO/cGMP signalling and neurotoxicity induced by voltage-gated sodium (Na_V_) channels. An optimized version of PnTx2-6 named PnPP-19 maintained beneficial erectile properties but lost activity against Na_V_s and hence its side-effects. PnPP-19 was also effective in treating pain *via* opioid and cannabinoid systems, and glaucoma *via* modulation of intraocular pressure. Another spider ICK peptide is HwTx-IV isolated from the tarantula *Cyriopagopus schmidti* that modulates Na_V_s and has analgesic properties (Xiao et al., 2008; Cardoso and Lewi, 2018; Cardoso and Lewi, 2019). Lopez et al. investigated HwTx-IV properties to inhibit Na_V_1.7, a potential analgesic target, and Na_V_1.6, a defined off-target, using synthetic peptide analogues. The ratio of Na_V_1.7/Na_V_1.6 inhibition was increased when the E^4^K mutation was combined to *R* ^26^A and Y^33^W, and by substituting the C-terminal amidation by a carboxylated motif. In contrast, this ratio was decreased by E^4^K alone, or by combined substitutions E^1^G/E^4^G or E^4^K/*R* ^26^Q. These results demonstrated it is possible to manipulate the selectivity of venom peptides towards specific Na_V_ subtypes to improve the potency and specificity.

Chronic pain, especially visceral pain, was shown treatable with ICK peptides inhibiting Na_V_s as reported for Tap1a and Tsp1a isolated from the tarantulas *Theraphosa apophysis* and *Trixopelma sp*, respectively (Cardoso et al., 2021; Jiang et al., 2021). Hu et al. evaluated the structure-function properties of Tap1a associated to potency and selectivity for Na_V_s, and to optimize Tap1a with greater *in vivo* effect. By incorporating residues from optimized NaSpTx1 peptides, optimized Tap1a peptides were designed with greater potency and selectivity for Na_V_ subtypes in chronic pain, as well as greater *in vivo* effects. Preclinical efficacy in treating several neurological disorders is also demonstrated by the ICK peptide Phα1β isolated from *P. nigriventer* (Antunes et al., 2021; Silva et al., 2021). Diniz et al. investigated how the analgesic effects induced by Phα1β and morphine modify the central nervous system (CNS) function. Phα1β and morphine analgesic effects have a different profile in the CNS, with Phα1β selectively inhibiting activity in the unilateral motor cortex and cingulate cortex, while morphine treatment led to small and selective inhibition of the bilateral amygdala striatum and accumbens.

Venoms from Araneomorphae spiders differ from Mygalomorphaes by the predominance of large proteins with enzymatic activity that enhances the envenomation process. The therapeutic and biotechnological potential of the venom of the Araneomorphae brown spiders of the genus *Loxosceles* was reviewed by Gremski et al. These venoms are known to induce dermo necrosis, oedema and haemorrhage that together are defined as loxoscelism. These symptoms are induced by phospholipases D, which have now been engineered to a non-toxic form to produce antivenom or a vaccine to prevent loxoscelism. Other components of these venoms include hyaluronidases, allergen factor, and ICK peptides, amongst others, which can be used as pharmacological tools, insecticides, or drug candidates to tackle cancer and pain.

Snake venoms comprise a range of proteins and peptides that induce hemotoxic, neurotoxic, and cytotoxic effects in snakebite victims (Ferraz et al., 2019). Vasconcelos et al. reviewed the structure-function relationships of snake disintegrins which modulate cell-cell and cell-matrix interactions. In this review, disintegrins were classified into seven groups based on disulphide pattern and sequence signatures, which facilitated identification of new disintegrins. Structural signatures associated to disintegrin-integrin interactions are presented to assist the understanding of their structure-function properties. Nicotine acetylcholine receptors (nAChR) are targets of snake peptides aimed at muscles to induce paralysis (Ferraz et al., 2019). Kasheverov et al. have developed snake peptides labelled with fluorophores to probe and visualize nAChR in cells and tissues. The 3FTX α-cobratoxin and the linear peptide azemiopsin from snake venom were labelled with a small synthetic analogue of green fluorescent protein (GFP) named *p*-HOBDI-BF_2_. The introduction of the synthetic GFP preserved the peptide affinity for nAChR while the full-length GFP induced considerable loss of affinity. Although useful in the visualization of nAChR in live cells, the use of synthetic GFP-labelled snake peptides was limited in rodent tissue and in flow cytometry applications.

GPCRs are the targets of almost 30% of drugs currently on the market. This way, studies of venom peptides that can modulate their activity with the purpose of drug development is of particular importance. We end this research topic with a review by Van Baelen et al. discussing venom peptides that target GPCRs and their structure-function properties. These GPCR-targeting venom peptides were divided into two classes: endogenous related agonist-mimicking peptides, and non-endogenous related peptides with agonist, antagonist and allosteric properties. This review highlights the absence of toxicity in some of these venom peptides that display high affinity and selectivity to GPCRs that can be largely exploited for therapeutic development, as well as the importance of using ecologically relevant animal models to decipher the biological role of these venom peptides.
